# “Dietary fibre”: moving beyond the “soluble/insoluble” classification for monogastric nutrition, with an emphasis on humans and pigs

**DOI:** 10.1186/s40104-019-0350-9

**Published:** 2019-05-24

**Authors:** Barbara A. Williams, Deirdre Mikkelsen, Bernadine M. Flanagan, Michael J. Gidley

**Affiliations:** The University of Queensland, QAAFI Centre for Nutrition and Food Sciences, St. Lucia campus, Brisbane, Qld 4070 Australia

**Keywords:** Cereal grains, Dietary fibre, Fruits, Large intestinal fermentation, Legumes, Microbial activity, Microbiota, Plant cell walls, Short-chain fatty acids, Vegetables

## Abstract

This review describes dietary fibres originating from a range of foods, particularly in relation to their plant cell walls. It explores the categorization of dietary fibres into “soluble” or “insoluble”. It also emphasizes dietary fibre fermentability, in terms of describing how the gastro-intestinal tract (GIT) microbiota respond to a selection of fibres from these categories. Food is categorized into cereals, legumes, fruits and vegetables. Mention is also made of example whole foods and why differences in physico-chemical characteristics between “purified” and “non-purified” food components are important in terms of health. Lastly, recommendations are made as to how dietary fibre could be classified differently, in relation to its functionality in terms of fermentability, rather than only its solubility.

## Introduction

Dietary fibre (DF) is considered essential for overall human health. Epidemiological studies have shown that diets which are high in fat, sugar, and salt, and low in DF (mostly associated with plant-based foods) can predispose the consumer to the many chronic diseases of our time, such as diabetes [1], obesity [2], cardio-vascular disease [3], certain cancers [4] and more [5]. Hence, the current interest by both nutrition professionals and the public for the inclusion of DF in a healthy diet.

DF is the main non-digestible component of monogastric diets, and is known to influence gastrointestinal tract physiology. There are three main mechanisms, whereby it is thought to have this influence. Firstly, by physical “structuring” of digesta, which is relevant to feelings of satiety and control of food intake [6]. Secondly, by modulation of digestive processes such as those which control transit time, which contribute to the control of circulating glucose and lipid levels [7], and lastly, by acting as an energy source for microbial fermentation, particularly (but not only) in the large intestine [8]. These mechanisms relate to characteristics such as dispersibility in water (water-holding capacity), viscosity, binding ability, absorptive capacity, faecal bulking capacity and fermentability [9, 10], which are summarized in Table 1.

**Table 1:** Dietary fibre- physico-chemical characteristics and relationships to gut effects (modified from [9])

DF characteristic	GIT effect	Systemic effect	References
Water-holding capacity & viscosity	Slows gastric emptying; Changes digesta mixing; Alteration of digestive enzyme activity; Stimulates passage rate;	Slows digestion, especially of protein and lipids; Associated with reduced plasma cholesterol; Blunting of glycaemic response	[11–13]
Bulking	Gastric distension; Changes in mixing & diffusion;	Decrease food intake;	[14, 15]
Adsorption of compounds (e.g. bile salts, polyphenols & minerals)	Increases bile acid excretion & other compounds; Retention of polyphenols until large intestine;	Blood cholesterol; fermentation of polyphenols;	[16, 17]
Encapsulation	Plant cell walls encapsulate e.g. starch granules;	Transport of starch (resistant) to LI for fermentation;	[18–21]
Fermentability	Increases microbial biomass & fermentation end-products (e.g. SCFA); Induces selection of specific microbes;	Energy for colonocytes; influences satiety; faecal bulking; “colonization resistance” to pathogens;	[22, 23]

In the past, it has been more usual to take a reductionist approach, and use either a purified form of DF such as cellulose [24–26] or various oligosaccharides [27–30] amongst others, and/or to examine the response of specific microbial species to purified fibre components [31, 32]. More recently, it is being realized that while pointing in useful directions for the determination of mechanisms by which DF can have its beneficial effects, particularly from a microbial perspective, it is also clear that these purified substrates, are not representative of food as consumed. Therefore, there is increasing literature, reporting work done with whole foods [18, 19, 33]. However, for this work to be useful, it will still be important for the test foods/feeds to be extensively characterised.

*In vitro* and *in vivo* methodologies can be used to test hypotheses examining effects of specific dietary components on physical, chemical and/or biological outcomes under controlled environmental conditions. *In vitro* methods usually involve laboratory-based mimics of one or more of the environments encountered in the digestive tract including: stomach [34], small intestine [35], and LI [36]. *In vivo* studies on the other hand, require the use of either animal models, or the test organism(s), and aim to disentangle mechanisms of action of specific dietary components. In that context, pigs are often used as a model for humans, as they share similar patterns of food intake [37], digestion patterns [38], and comparable (though not identical) gut microbiology [39, 40]. Ultimately, the best approach would be to integrate findings from epidemiology*, in vivo*, *in vitro,* and clinical studies, to obtain a comprehensive overview of the mechanisms and effects of dietary components such as DF, on final health outcomes [7].

The emphasis of this review, is how the gastro-intestinal tract (GIT) microbiota responds to a selection of compounds from the categories of “soluble” and “insoluble” DF. Mention will also be made of example whole foods and why differences in physico-chemical characteristics between “purified” and “non-purified” food components are important in terms of health. The focus will be on work from both porcine and human studies as relevant. Lastly, recommendations will also be made as to how DF could be classified differently, in relation to its functionality in terms of fermentability, rather than only its solubility.

### Dietary fibre - definition, classification, and sources

At least since the beginning of the twentieth century [41, 42] “crude fibre” has been used to describe the plant-derived component of feed and foods, which was resistant to digestion by mammalian enzymes (particularly in animal nutrition). By the 1950’s, the term “dietary fibre” was adopted, particularly when referring to human nutrition (e.g. [43]). Since then, there have been many definitions, as reviewed by Jones [44]. A workable definition derived from this author ([44] is: “dietary fibre is an overall description of mainly carbohydrate polymers derived from or contained by (usually) edible plants, (ranging from DP >3 to >10) which are neither absorbed within the small intestine, nor hydrolysable by mammalian digestive enzymes in the small intestine”. In general, this includes celluloses, hemicelluloses, lignins, oligosaccharides, pectins, gums and waxes, as well as resistant starches, resistant proteins, and associated compounds such as polyphenols [9]. In the official definitions listed by Jones [44] further qualifications are made according to physiological effects, and for guidance for the food industry.

DF has been categorized according to: source, solubility, fermentability, and physiological effects [10]. In terms of methods used for the quantification of fibre within feeds/foods, there have been many methods described for both animal [45] and human nutrition [46, 47], though there is often controversy as to the “best” method for purpose [48–50].

One simple classification which is commonly used, is to differentiate between “soluble” and “insoluble” fibres [10], based on the ability to be fully dispersed when mixed with water [9]. However, polysaccharides classified as “soluble” may be quite variable in their actual solubility in water [51]. Both soluble and insoluble DF share many physical properties including water-binding capacity, and capacity to bind mineral cations [9]. Their fermentability however, can vary according to the physico-chemical properties of each compound [52].

The “soluble” classification of DF typically includes compounds such as hemicelluloses (e.g. xyloglucans, galactomannans mixed-linkage glucans), pectins, gums and mucilages. On the other hand, cellulose, lignin, and resistant starch are considered to be examples of insoluble DF [9]. However, depending on the plant source and degree of post-harvest processing, many of these polymer types can be either soluble or insoluble. All of these fibres differ in their monosaccharide components and the glycosidic linkages that connect them together as shown in Table 2.

**Table 2:** Structural composition of different dietary fibres (note- “soluble” may indicate “partially soluble” (modified from [9])

DF	Solubility	Main unit	Branch units	References
Cellulose	Insoluble	β- (1,4) Glucose	–	[53]
Lignin	Insoluble	Polyphenols	Polyphenols	[54]
Resistant starches	Insoluble	Helical amylose	1,6 glucose in amylopectin	[55]
Mix-linkage glucans	Soluble	β- (1,3) glucose	–	[56–58]
		β- (1,4) glucose	–	
Hemicelluloses Arabinoxylan Xyloglucan Galactomannans	Soluble Soluble Soluble	Xylose Glucose Mannose	Arabinose Xylose Galactose Glucose	[56]
Pectins	Soluble	Galacturonic acid with methoxy groups	Arabinose Galactose	[59]
Gums Guar Agar	Soluble Soluble	β −1,4-linked mannose *D*-galactose & (3,6) anhydro-L-galactose	Galactose -	[60]
Non-digestible oligosaccharides Fructooligosaccharide Galactooligosaccharide	Soluble Soluble	*D*-fructose residues Galactose, with terminal glucose unit.	- -	[61, 62]

In terms of nutritional guidelines, “dietary fibre” is often considered as a single entity. However, from a physico-chemical perspective, this one term is known to include a wide range of different materials. These vary substantially in their biological and chemical properties, not only within the plant, but also upon consumption and behaviour within the GIT.

### Plant foods as sources of dietary fibre

Plant cell walls (PCW) are essential to maintain plant structure and function [8]. They are rich in a range of polysaccharides and are present in all plant-based foods, though with different structure and chemistry, depending on the source (fruit, vegetables, legumes and cereals) [63]. In terms of human nutrition, all of these plant-based foods are highly relevant. For pigs fed under commercial production conditions, the cereals and legumes are most immediately relevant, though there is increasing interest in using feeds originating from fruit and vegetable wastes [64]. The amounts and relative proportions of all of the PCW components vary depending on botanical source, as well as origin, function and maturity of the plant tissue [65].

### Fruits and vegetables

In human dietary recommendations around the world, fruits and vegetables are recommended to form a substantial part of the daily diet, given their known health-promoting properties.

There are many epidemiological studies which have shown a beneficial link between high fruit and vegetable intake, and improved health outcomes [66–68]. Not only are they a rich source of a variety of DF, containing varying proportions of non-fermentable, slowly and rapidly fermentable fibres, they also contain a range of polyphenolic compounds, and essential vitamins and minerals.

Generally, fruits contain mostly sugars and DF such as pectin. For example, in addition to dietary fibre (Table 3), apples contain 6% fructose and 3% sucrose [69] that are typically available for digestion in the small intestine. Vegetable foods on the other hand, vary more in terms of their plant origin than fruits, including leaves, stems, roots and tubers which vary not only in their DF content and proportions, but also in terms of their protein, and secondary metabolite contents [69]. Table 3 shows a range of fruits and vegetables indicating the variability of the DF content.

**Table 3:** Dietary fibre content of selected fruits and vegetables (modified from NUTTAB, Food Standards Agency Australia^a^)

Fruits	Total DF, g/100g dry matter	Vegetables	Total DF, g/100g dry matter
Apple, with peel	16.7	Beetroot	26.7
Cavendish banana	10.1	Broccoli	34.0
Cherries	8.7	Cabbage, white	30.0
Grapefruit	14.5	Carrot	34.2
Mango	9.4	Celery	29.4
Orange	18.0	Cucumber	15.8
Peach	16.1	Iceberg lettuce	33.3
Pear	21.2	Sweetcorn	22.0
Pineapple	13.6	Tomato	20.7
Strawberry	31.6	Zucchini	23.1
Watermelon	6.4		

^a^Data from Food Standards Agency Australia NUTTAB Release 2010, Last accessed April 19, 2019 from: http://www.foodstandards.gov.au/science/monitoringnutrients/nutrientables/nuttab/Pages/default.aspx

Processing (such as cooking, drying, chopping, or blending) of fruits and vegetables can lead to significant changes in the DF content of these foods both in terms of amounts and functionality. For example, peeling will most likely decrease the DF content, while cooking may actually concentrate the DF content [69]. However, the type and proportion of different fibres within the original material will have the most influence on its functionality in the gut. So, while many fruits and vegetables are thought to ferment rapidly and may therefore contribute less to faecal bulking than less fermentable fibres [10], this may be offset by the resultant increase in bacterial numbers [70].

### Cereals and legumes

Cereal grains are the most widely consumed, and an important source of energy in global nutrition, both of humans and monogastric production animals. “Whole grains” most commonly refer to all components of the cereal grain, including the endosperm, aleurone, and pericarp [8] from cereal crops such as rice, wheat, maize, oats, sorghum, and rye. The DF components of cereal grains include cellulose, and hemicelluloses such as arabinoxylan and mixed-linkage glucans [71].

Globally, legumes are an important source of protein both in human and animal nutrition. They also provide energy in the form of carbohydrates, DF, lipids (for leguminous oilseeds) as well as some minerals and vitamins [72]. However, the presence of secondary plant metabolites (anti-nutritional factors) has been perceived as having a negative influence on digestibility and final energy utilization [73]. Their use in animal feeding has increased considerably since the ban, imposed by the European Commission in 2001, of all animal-based products in animal feeding [73]. In terms of pig production, the DF content of legumes has been reported as leading to a reduction in digesta passage rate, and a lower feed intake [74]. The forms of DF present in legumes can include cellulose, and hemicellulose such as oligosaccharides including those of the raffinose family [73].

Table 4 shows a range of cereals and legumes illustrating the variability of the total DF content within this food group.

**Table 4:** Dietary fibre content of selected cereals & legumes (modified from NUTTAB, Food Standards Agency Australia^a^)

Cereals	Total DF, g/100g dry matter	Legumes	Total DF, g/100g dry matter
Barley, pearled, raw	11.7	Haricot beans	20.8
Millet, raw	9.3	Lentils	15.3
Oats, rolled	9.5	Lima beans	19.6
Rice	3.2	Peas, green, raw	25.6
Wheat flour, wholemeal	11.3	Red kidney beans	21.5
		Soybeans	20.1

^a^Data from Food Standards Agency Australia NUTTAB Release 2010, Last accessed April 19, 2019 from: http://www.foodstandards.gov.au/science/monitoringnutrients/nutrientables/nuttab/Pages/default.aspx

### Gut microbiota- activities and communities

The GIT microbiota includes the entire microbial population within the GIT, from the mouth to the anus. It includes bacteria, fungi, viruses and archaea, though most studies have focussed on the bacteria as, until now, they have been considered to be most active [75]. In monogastrics, the main site of fermentation is considered to be the LI [76], though it is slowly being recognized that while microbial numbers and activity are less in the stomach and small intestine, the activity occurring here is also likely to be relevant for overall host health [77]. Any partial gastric or small intestinal fermentation also has the potential to alter the course of subsequent fermentation in the LI.

The human GIT bacterial community has been classified into at least seven phyla, of which four are predominant (usually ~ 98% of the total population). These are the Firmicutes (58–88%), Bacteroidetes (8.5–28%), Proteobacteria (0.1–8%), and Actinobacteria (2.5–5%) [78]. However, bacterial community profiling from faeces has shown that as many as 60% of bacterial species are not yet identified, [78, 79]. Sommer et al. [80], provides an excellent perspective on how the human intestinal microbiota “resilience” is critical in influencing health and disease states, particularly discussing this concept with regards to diet, antibiotic or bacteriotherapy-induced perturbations. Furthermore, efforts continue to be made to develop ways to describe the complex gut microbial landscape across large human populations and geographies, where the term entrotypes is once again being revisited and refined by standardising and controlling the sample processing and data analysis, as well as providing functional, ecological and medical contexts [81].

### Fermentation of dietary macronutrients

Dietary components remaining undigested at the end of the small intestine can potentially be fermented within the LI. Ideally, a wide range of fermentable carbohydrates present in the diet can provide both nutritional and potential health benefits. These include: regular bowel movements, competition of active bacteria against potentially pathogenic organisms [82], stimulation of potentially beneficial bacteria [7], production of end-products such as SCFA, and prevention of protein fermentation, thus avoiding production of potentially toxic and cancer-promoting metabolites [83]. The full complexity of the gut microbiota and all of its functions, as well as its effect on its host organism, is only beginning to be understood, but it is clear that DF, in all its forms, is essential for a healthy digestive tract and host, and that a significant part of this benefit is microbially-mediated.

#### Carbohydrates

Bacterial utilisation of fermentable carbohydrates results predominantly in the production of SCFA such as acetic, propionic and butyric acids, but a range of other carboxylic acids can also be produced, including lactic acid [76]. These end-products are generally beneficial for GIT health [84, 85]. Once produced, the SCFA can have multiple effects within humans and other mammals, and are heavily utilised as a source of energy, by both humans [86] and bacteria [87].

Acetic, propionic and butyric acid consist of two, three, and four carbon atoms, respectively, and are the principal products of carbohydrate fermentation by bacteria in the GIT [88]. Within the LI, SCFA are important promoters of colonic health as they are involved in the control of colonic mobility, colonic blood flow and GIT pH, all of which affects nutrient and electrolyte absorption [76, 89].

Acetic acid is the predominant SCFA in venous blood [88]. Acetic acid produced in the LI is absorbed across the GIT epithelium wall into the portal vein, and diffuses through the peripheral venous system [76]. It has also been shown to be the principal SCFA fermentation product of pectin and xylan in the GIT [90]. In addition, there is evidence to indicate its interaction with the G protein-coupled FFAR2 receptor which impacts inflammation and the immune response [91].

Although propionic acid can be metabolised from a range of substrates, including proteins, the most common metabolic pathway involves fermenting carbohydrates [92]. Propionic acid is absorbed into the portal vein and moves to the liver where it can be metabolised by hepatocytes [76]. Approximately 90% of propionic acid absorbed into the portal vein is metabolised in the liver, of which a substantial proportion is used for gluconeogenesis [88], as well as interacting with the immune system through the FFAR2 receptor [65, 93]. There are also suggestions that propionate can alter cholesterol synthesis [94]. It has also been shown to stimulate feelings of satiety, thus influencing food intake [92].

Butyric acid is a major oxidative fuel for colonocytes (colonic epithelial cells), supplying approximately 60–70% of their energy requirements [86]. Associated with this function, it has been shown that butyrate influences metabolic pathways of the gut by changing cellular growth and metabolism [94]. By this means, it is thought that butyric acid is involved in the prevention of colonic cancer [95, 96].

#### Proteins

Protein fermentation refers to the bacterial breakdown of proteins to amino acids, as well as their further breakdown to ammonia and other potentially toxic compounds such as indoles, phenols, and amines [97]. This process normally increases when there is a shortage of fermentable carbohydrates available to the gut bacteria as a source of energy. Health benefits of reduced protein fermentation are related to the reduction of ammonia and other nitrogenous, phenolic and sulphurous compounds in the GIT [98], while increased protein fermentation is considered to be detrimental to GIT health [99].

Ammonia (NH_3_) is the dominant by-product of the fermentation of amino acids in the GIT. Excess protein fermentation can lead to an increase of NH_3_ and amines. NH_3_ then moves from the GIT into the bloodstream and is detoxified in the liver or muscles, with a large amount converted to urea and excreted by the kidneys [100]. Protein fermentation can also lead to end-products such as branched-chain SCFA, amines, phenols, sulphides and thiols [94]. With the exception of branched-chain fatty acids, excessive production of these metabolites has been linked to several bowel disorders, including colon cancer [101, 102] and Crohn’s disease [103]. However, if there is a constant supply of carbohydrates and sufficient saccharolytic bacteria, the detrimental effects of these metabolites can be significantly reduced [94].

### Dietary fibre fermentability- physico-chemical effects

The molecular structure of individual forms of purified DF, the matrix structure, and the particle size of DF can all affect its availability for bacterial enzymes and the ability of specific bacterial species to colonize and/or invade fragments of plant tissues [26, 33, 104, 105]

### Molecular structure

Dietary fibre includes a wide range of mostly carbohydrate polymers ranging from soluble polymers (such as pectins and various oligosaccharides) to insoluble ligno-cellulosic materials and resistant starch [106] as discussed previously. Basically, these compounds comprise varying numbers of monosaccharide units joined by glycosidic linkages. They differ according to the composition of the monosaccharides, the types of linkages, and the presence (or not) of branches on the backbone structure [107]. From a nutritional perspective, Kumar et al. [107] have summarised the non-starch polysaccharide (NSP) molecules and their structures present within plants.

### Soluble DF

The solubility of polymers depends on several different factors and molecular properties, such as the conformational entropy [51]. Many polymers while categorized as being “soluble” are actually poorly soluble in water, and can either aggregate or phase-separate over time [51]. This self-association tendency is strongest where the polymers can form side-by-side ribbon binding or co-axial multi-stranded helices, and tends to be more prevalent with less backbone substitution. Broadly speaking, solubility of polymers seems to improve as polymer molecular structures become: (i) more branched and with a greater diversity of linkages, or (ii) smaller. High molecular weight coupled with solubility results in thickening of solutions [51]. Within the soluble DF, there are known to be substantial differences in their fermentabilities, with many of them promoting the proliferation of health-promoting bacterial species such as *Bifidobacterium*, *Lactobacillus*, and *Eubacterium* [108].

**Fig. 1:**
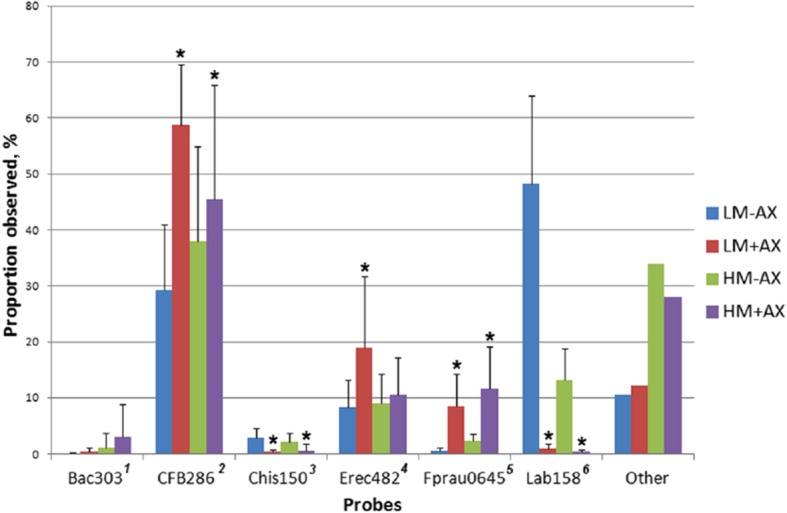
Caecal bacterial proportional counts (%) of probe versus diet as observed using FISH. The diets tested were LM-AX (Low meat- no added arabinoxylan; LM + AX- low meat with added AX; HM-AX- high meat no added AX; HM + AX- high meat with added AX, as described previously [22]. * indicates a significant change (*P* < 0.05) in the proportion of bacteria observed using the stated probe as a result of the introduction of AX to the diet, as calculated using a non-parametric Mann-Whitney test. ^1^ targets 64% of the order Bacteroidales; ^2^ targets most members of the genus *Tannerella* and the genus *Prevotella* of the class Bacteriodetes; ^3^ targets most of the *Clostridium histolyticum* group (*Clostridium* cluster I and II); ^4^ targets most of the *Clostridium coccoides*-*Eubacterium rectale* group (*Clostridium* cluster XIVa and XIVb); ^5^ targets

In an *in vivo* study where pigs were fed two levels of BBQ meat (LM and HM), with and without the addition of AX (−AX and + AX) [22], fluorescence *in situ* hybridization (FISH) indicated that the presence of soluble fibre altered the caecal bacterial proportional counts as shown in Fig. 1. These data suggest that the presence of AX led to a significant shift in the microbiota in the presence of a soluble DF.

Purified soluble oligosaccharides have become very popular as potential prebiotics [109] partly because they do not alter the viscosity or texture of foods due to their low molecular weight, and because they are usually highly fermentable. However, they may be so readily fermentable that they may be completely utilized by the end of the terminal ileum [61]. It is to be recommended therefore, that they be fed in conjunction with more slowly fermentable DF, which can allow carbohydrate fermentation to continue in the LI [99]. Oligosaccharides are also found normally in many plant tissues in the form of fructans [51]. Plant foods known to contain fructans include cereal grains, onions, chicory, and Jerusalem artichoke.

Mixed-linkage β-glucans are non-cellulosic polymers which occur extensively in cereal grains, particularly barley, oats and rye [107]. They are generally known to be soluble [110], and are readily fermented by the GIT microbiota [106]. Arabinoxylans on the other hand, are heteroxylans which are abundantly present in the PCW of cereals and grasses, particularly wheat, and also within the genus *Plantago* [107]. Arabinoxylans are generally highly viscous in aqueous solutions. It is also considered to be highly fermentable as has been shown *in vitro* [106] using an inoculum of pig faeces.

Pectins are structural polysaccharides present within the primary cell walls of many fruits and vegetables, which are extractable into a soluble, viscous form. They have an extremely diverse structure, sharing some common features such as the presence of galacturonic acid in the polysaccharide backbone [51]. Previous *in vitro* studies using pig faeces have shown pectin to be highly fermentable, both in the presence of chyme [111] and also using both adult and unweaned piglet faeces [112].

### Insoluble DF

**Fig. 2:**
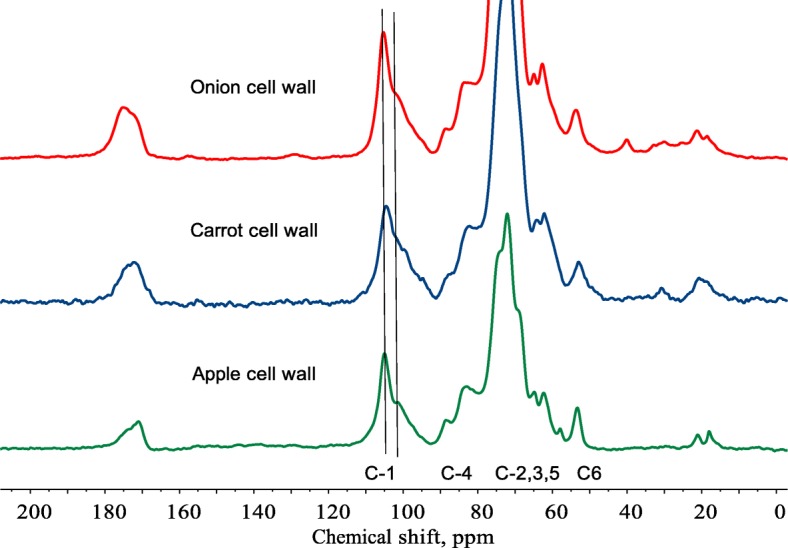
^13^C CP/MAS NMR of onion, carrot and apple cell walls. All spectra are from samples with added water. The region from 95 to 110 ppm is the most diagnostic for polysaccharides as this is the region where the anomeric carbons (C-1) are seen. The peak at 105 ppm is typical of cellulose and the shoulder at 99 ppm is expected for C-1 of galacturonic acid. The presence of cellulose is also clear from the C-4 peak at 90 ppm. The fact that otherwise soluble galacturonic acid from pectin can be seen in the CP/MAS (‘solid state’) spectrum of wet cell walls, suggests that the pectin is bound to cellulose rendering it insoluble

Cellulose is a major structural component of PCW from almost all plant foods. It is a linear polymer of glucose units linked by β-(1-4) linkages. It is highly insoluble in water, and cannot be degraded by human digestive enzymes, but is fermented to varying extents by gut bacteria particularly in ruminant animals [113], and also in pigs [25, 114], and humans [115–117]. Within plant cell walls, cellulose is also cross-linked with otherwise soluble pectin or hemicelluloses, rendering them insoluble. Using ^13^C CP/MAS NMR of wet cell wall isolates from apples, carrots and onions, it is possible to identify the presence of both pectin (galacturonic acid C-1, 99 ppm) and cellulose (C-1105 ppm) in a relatively rigid form, consistent with the two components interacting. The isolated plant cell walls from apples, carrots and onions contain cellulose and a fraction of pectin that cannot be removed by washing and is therefore insoluble, as shown in Fig. 2.

The cell walls of many plants are also classified as insoluble, and vary greatly in their ability to be fermented. At one extreme, the soluble and insoluble fractions of refined cereal flours or food products made from them, had essentially identical *in vitro* fermentation behaviour with a porcine faecal inoculum [118]. Both fractions were mostly composed of AX, and while the insoluble fraction was difficult to extract, both had comparable fermentation characteristics once extracted. At the other extreme, the fibrous vascular tissue present in e.g. mangoes resists *in vitro* fermentation even after all of the more fleshy tissue around it (also "insoluble") has been fermented [18]. A further example of insoluble fibre is resistant starch from certain uncooked starch granules [19]. Another type of resistant starch is that held within plant well walls. For example, starch within cells in banana, were slow to ferment as they were unavailable until the cell walls surrounding it had been fermented [18].

There are therefore examples of insoluble DF that are rapidly fermented (e.g. from refined flour), slowly fermented (e.g. resistant starch, wheat bran) or essentially not fermented (e.g. vascular tissue). This provides clear evidence that equating insoluble fibre with non-fermentable fibre is no longer a valid premise.

### Effects of processing

#### Fractionation

Modifications of some properties of DF may occur at the stage of mechanical processing such as the dehulling and milling of cereals [51] to make flour. Milling disrupts cell wall structure and alters particle size [51].

For example, wheat contains various proportions of NSP including arabinoxylans and β-glucans, which are enriched during the milling process to produce flour for human consumption [119]. In terms of pig production, it is often wheat by-products, such as wheat bran, and wheat middlings which are important components of the diet. Both of these products are higher in DF than the extracted flour [119]. Wheat bran comprises the pericarp and the aleurone layer of the grain, and constitutes roughly 10% of the total weight of the wheat ground to flour. It is known to have a high level of insoluble lignified fibre, which is generally resistant to fermentation in the LI [120].

#### Cooking (baking, toasting, roasting, extrusion etc.)

It is well known that the chemical structure of starches can be markedly altered by heat treatments [51]. Additionally, cooking of plant tissues can also alter physical and chemical properties of PCW, such as cell separation and dissolution of the middle lamella, breakdown of pectins, and formation of cross-links between food components [121]. Extrusion cooking has been shown to actually break PCW bonds, reducing insoluble fibre content and increasing soluble fibres [121].

#### Plant tissue structure- the effect of “whole” foods

Raw plant tissues usually retain much of their cell-level integrity following mastication [121]. Consequently, there will be less breakdown of PCW in the small intestine, and digesta viscosity will be lower, and less cell contents will be available for mammalian digestion. However, upon reaching the LI, microbial fermentation can lead to a breakdown of the PCW, and consequent release of the cell contents for further fermentation.

McDougall et al. [121] in an excellent though now dated review, described this is as a “sequential stripping away” of components from the PCW, whereby the PCW components have different roles to deliver the ultimate beneficial effects of overall DF.

**Fig. 3:**
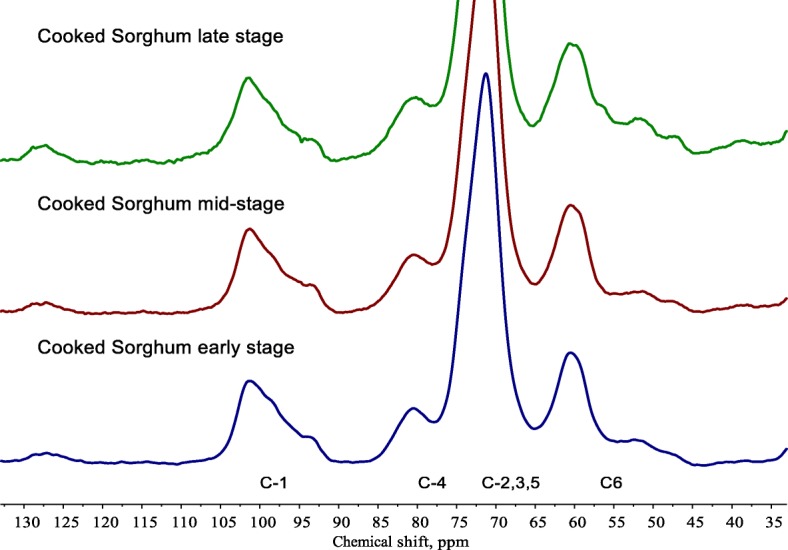
^13^C CP/MAS NMR spectra of cooked sorghum at early, mid- and late stages of *in vitro* fermentation. The spectrum of cooked sorghum is dominated by starch. The anomeric carbon (C-1 region) from 90 to 105 ppm is used to calculate the starch molecular order

For example, an *in vitro* study compared fermentability of chewed banana and mango tissue, and showed that differences in physical characteristics of the two plant tissues led to profound differences in the fermentability. While thick cellulosic vascular structures remained for the mango post-fermentation, the banana showed significant release of the entrapped starch granules, after the breakdown of the PCW (from 0 to 48 h) [18], though these still remained intact by 48 h. In the study by Warren et al gelatinised starch within cell walls of cooked sorghum grains was still observed at the late stage of *in vitro* fermentation. Using solid state ^13^C CP/MAS NMR it is possible to calculate the level of starch molecular order or crystallinity. The molecular order was unchanged throughout the fermentation [19], as shown in Fig. 3.

In contrast, for carrots which were processed to obtain different particle sizes, larger particles (cell clusters) resulted in faster production of gas, and increased concentrations of SCFA after fermentation *in vitro* with a porcine faecal inoculum [33]. At least two possibilities could explain this. Firstly that junctions between cells, allowed bacteria to attach more readily to cells, allowing better access to the PCW, or secondly, that in the smaller particles, pectin between cells had been lost, and so this fraction was no longer available to be fermented. Further studies are required to elucidate the mechanism responsible.

## Conclusions

This review describes different DF, particularly those categorized as being “soluble” or “insoluble”. The emphasis has been on work describing how the GIT microbiota (e.g. from pigs) responds to a selection of compounds in these categories. Some of the characteristics of DF functionality arise directly from their molecular structure as determinants of the tendency to self-associate (simpler, less-branched structures) or ferment slowly (complex, more-branched structures). However, much DF in both food and feed is in the form of plant tissue pieces. In this case, the cellular structure results in both insoluble DF and encapsulation of cellular components, sufficient to prevent digestion and absorption in the small intestine. This phenomenon provides a mechanism for intracellular contents such as starch, protein and secondary metabolites to be made available for fermentation in the LI after passing through the SI intact. Purified DF, such as oligo- or polysaccharides extracted from whole plant foods, are not necessarily representative of those whole foods, but do provide insights into potential mechanisms by which DF has its beneficial effects in the gut.

The classification of potentially fermentable carbohydrates into soluble and insoluble, while helpful, is no longer enough for the information required to elucidate mechanisms by which DF has beneficial effects on monogastric health. Characteristics such as fermentability (including both kinetics of fermentation and end-products) will undoubtedly make a significant contribution to our understanding of how plant-based foods/feeds affect overall health in humans and pigs.

## Abbreviations

DF: Dietary fibre
GIT: Gastrointestinal tract
LI: Large intestine
NH_3_: Ammonia
NSP: Non-starch polysaccharides
PCW: Plant cell walls
SCFA: Short-chain fatty acids
SI: Small intestine
